# Metagenomic Analysis of Fecal Archaea, Bacteria, Eukaryota, and Virus in Przewalski's Horses Following Anthelmintic Treatment

**DOI:** 10.3389/fvets.2021.708512

**Published:** 2021-08-18

**Authors:** Dini Hu, Jianming Yang, Yingjie Qi, Boling Li, Kai Li, Kai Meng Mok

**Affiliations:** ^1^School of Ecology and Nature Conservation, Beijing Forestry University, Beijing, China; ^2^Xinjiang Research Centre for Breeding Przewalski's Horse, Urumqi, China; ^3^Xinjiang Kalamaili Ungulate Nature Reserve Management Center, Changji, China; ^4^China National Environment Monitoring Centre, Beijing, China; ^5^Department of Civil and Environmental Engineering, University of Macau, Macao, China

**Keywords:** przewalski's horse, anthelmintic treatment, intestinal microbiota, metagenomic sequencing, functional annotation

## Abstract

Intestinal microbiota is involved in immune response and metabolism of the host. The frequent use of anthelmintic compounds for parasite expulsion causes disturbance to the equine intestinal microbiota. However, most studies were on the effects of such treatment on the intestinal bacterial microbes; none is on the entire microbial community including archaea and eukaryotic and viral community in equine animals. This study is the first to explore the differences of the microbial community composition and structure in Przewalski's horses prior to and following anthelmintic treatment, and to determine the corresponding changes of their functional attributes based on metagenomic sequencing. Results showed that in archaea, the methanogen of *Euryarchaeota* was the dominant phylum. Under this phylum, anthelmintic treatment increased the *Methanobrevibacter* genus and decreased the *Methanocorpusculum* genus and two other dominant archaea species, *Methanocorpusculum labreanum* and *Methanocorpusculum bavaricum*. In bacteria, *Firmicutes* and *Bacteroidetes* were the dominant phyla. Anthelmintic treatment increased the genera of *Clostridium* and *Eubacterium* and decreased those of *Bacteroides* and *Prevotella* and dominant bacteria species. These altered genera were associated with immunity and digestion. In eukaryota, anthelmintic treatment also changed the genera related to digestion and substantially decreased the relative abundances of identified species. In virus, anthelmintic treatment increased the genus of unclassified_d__*Viruses* and decreased those of unclassified_f__*Siphoviridae* and unclassified_f__*Myoviridae*. Most of the identified viral species were classified into phage, which were more sensitive to anthelmintic treatment than other viruses. Furthermore, anthelmintic treatment was found to increase the number of pathogens related to some clinical diseases in horses. The COG and KEGG function analysis showed that the intestinal microbiota of Przewalski's horse mainly participated in the carbohydrate and amino acid metabolism. The anthelmintic treatment did not change their overall function; however, it displaced the population of the functional microbes involved in each function or pathway. These results provide a complete view on the changes caused by anthelmintic treatment in the intestinal microbiota of the Przewalski's horses.

## Introduction

*Gasterophilus* spp. (horse botfly) is a common parasite in equids (1, 2). Their eggs and larvae can survive in the digestive system (e.g., stomach and intestine) of a host for 8–10 months (3, 4). Infection of horse botfly can cause serious clinical diseases, such as dysphagia, gastric and intestinal ulceration, gastric obstruction, and volvulus; it could even lead to severe risks of anemia, diarrhea, gastric rupture, peritonitis, perforating ulcers, and other complications (5–7). The horse botfly epidemic has been serious in the desert steppe of Xinjiang, China, with six species including *G. haemorrhoidalis, G. inermis, G. intestinalis, G. nasalis, G. nigricornis*, and *G. pecorum* commonly found in the local equids (8, 9). In botfly infection in Przewalski's horses, there is particular severity with a 100% infection rate, and an infection level much higher than other equine animals (10). At present, deworming is performed annually in winter through administration of anthelmintic compounds to control the infestation (9). However, the frequent use of these drugs will inevitably lead to drug resistance of parasites, which have been widely reported (11–13). It could also disturb the balance of the intestinal microbial community after removal of the parasites (14).

The equine gut hosts a complex microbial ecosystem with a variety of commensal, symbiotic, and pathogenic microbes. Disturbances to the normal intestinal microbiota could exert critical impact on the host's physiology. In horses, some disturbances are found related with colic (15, 16), diarrhea (17), obesity (18), and other clinical diseases. It is known that many factors such as nutrition and management, medication, age, disease, stress, and gender can influence equine intestinal microbiota (19, 20). However, studies on the effects of anthelmintic treatment for parasite expulsion on the intestinal microbiota of horses are still limited. Goachet et al. (21) were the first to report a reduction in cellulolytic bacteria and an increase in *Lactobacilli* and *Streptococci* in horses after anthelmintic treatment. Peachey et al. (22) found that bacterial phylum TM7 was reduced 14 days after anthelmintic treatment, while *Adlercreutzia* spp. were increased only 2 days after. Crotch-Harvey et al. (23) detected temporal differences of the bacterial community when horses were treated with anthelmintic drugs. Walshe et al. (24) found that the alpha and beta diversity of the bacterial community decreased at day 7 of post-anthelmintic treatment and reverted on day 14. Peachey et al. (25) confirmed again that anthelmintic treatment was associated with alteration of the relative abundances of the bacterial community. Daniels et al. (26) observed that anthelmintic treatment increased the relative abundances of *Deferribacter* spp. and *Spirochaetes* spp. For Przewalski's horses, Hu et al. (4) found that the removal of horse botflies through anthelmintic treatment decreased the alpha diversity of the gut bacterial community, increased its *Firmicutes* to *Bacteroidetes* (F/B) ratio, and increased the genera of *Streptococcus* and *Lactobacillus* and some pathogenic bacteria. Nonetheless, these studies only explored the changes of the intestinal bacterial community associated with anthelmintic treatments. There is no study available on the changes of the entire microbial community and its functional prediction due to anthelmintic treatment. In fact, fungi, viruses, and some other species also play vital roles in the physiology and immune system of the host (27); e.g., fungi of *Aspergillus, Candida, Fusarium, Penicillium*, and *Saccharomyces* and archaea of methanogens represent notable members of the intestinal microbiota (28, 29). Hu et al. (30) suggested that anthelmintic treatments on Przewalski's horses could impact the fungal communities even more than the bacterial communities. Therefore, metagenomic sequencing is used in this study to characterize the entire intestinal microbial community (archaea, bacteria, eukaryota, and virus) of Przewalski's horses prior to and following anthelmintic treatments (ivermectin), so as to identify the changes in microbial diversity and richness, as well as genes, functions, and metabolism pathways. The results will lead to a better understanding on the relationships between anthelmintic treatment and the equine intestinal microbiota.

## Materials and Methods

### Ethics Statement

This study was carried out in accordance with the recommendations of the Institute of Animal Care and the Ethics Committee of Beijing Forestry University. The Ethics Committee of Beijing Forestry University approved the experimental protocol. The management authority of the Kalamaili Nature Reserve (KNR) in Xinjiang approved the collection of Przewalski's horse fecal samples.

### DNA Extraction and Metagenomic Sequencing

In a previous study of the present research group (4), fecal samples of seven adult Przewalski's horses (four male, three female) of similar body weight in the KNR prior to (PATPH) and following (FATPH) anthelmintic treatment of ivermectin were collected for DNA extraction and 16S rRNA sequencing. The numbers of horse botfly larvae in the fecal samples of the FATPHs were also recorded for assessing their parasitic infection status before treatment. In this study, shotgun metagenomic sequencing was conducted on the same DNA extracts used for 16S rRNA sequencing in Hu et al. (4). Out of the seven pairs of DNA samples, three pairs were chosen for this study based on their corresponding high to low and in between total fecal larva counts (FATPH3: 2,966, FATPH6: 1,928, FATPH1: 724). The concentration and purity of these six DNA samples (PATPH3, PATPH6, PATPH1; FATPH3, FATPH6, FATPH1) were tested by TBS-380 and NanoDrop 2000, respectively. DNA extract quality was checked with 1% agarose gel.

The DNA extract was fragmented to an average size of about 400 bp using Covaris M220 (Gene Company Limited, Beijing, China) for paired-end library construction with NEXTFLEX Rapid DNA-Seq (Bioo Scientific, Austin, TX, USA). Adapters containing the full complement of sequencing primer hybridization sites were ligated to the blunt end of fragments. Paired-end sequencing was performed on an Illumina sequencing platform at Majorbio Bio-Pharm Technology Co., Ltd. (Shanghai, China) according to the manufacturer's instructions (www.illumina.com). Sequence data associated with this study have been deposited in the NCBI Short Read Archive database (BioProject ID: PRJNA722063).

### Sequence Quality Control and Genome Assembly

Data were analyzed on the free online Majorbio Cloud Platform (www.majorbio.com). The paired-end Illumina reads were trimmed of adaptors. Low-quality reads (length <50 bp or with a quality value <20 or having N bases) were removed by fastp (31) (https://github.com/OpenGene/fastp, version 0.20.0). Reads were aligned to the Przewalski's horse genome (GenBank accession no. GCA_000696695.1) by BWA (32) (http://bio-bwa.sourceforge.net, version 0.7.9a). Any hit associated with the reads and their mated reads were removed. Metagenomics data were assembled using MEGAHIT (33) (https://github.com/voutcn/megahit, version 1.1.2), which makes use of succinct de Bruijn graphs. Contigs with the length being or over 300 bp were selected as the final assembling results; they then were used for further gene prediction and annotation.

### Gene Prediction, Taxonomy, and Functional Annotation

Open reading frames (ORFs) from each assembled contig were predicted using MetaGene (34) (http://metagene.cb.k.u-tokyo.ac.jp/). The predicted ORFs with length 100 bp and over were retrieved and translated into amino acid sequences using the NCBI translation table (http://www.ncbi.nlm.nih.gov/Taxonomy/taxonomyhome.html/index.cgi?chapter=tgencodes#SG1). A nonredundant gene catalog was constructed using CD-HIT (35) (http://www.bioinformatics.org/cd-hit/, version 4.6.1) with 90% sequence identity and 90% coverage. Reads after quality control were mapped to the nonredundant gene catalog with 95% identity using SOAPaligner (36) (http://soap.genomics.org.cn/, version 2.21), and gene abundances in each sample were evaluated.

Representative sequences of nonredundant gene catalog were aligned to the NCBI NR database with an *e*-value cutoff of 1e^−5^ using Diamond (37) (http://www.diamondsearch.org/index.php, version 0.8.35) for taxonomic annotations. Cluster of orthologous groups of proteins (COG) annotation for the representative sequences was performed using Diamond against the eggNOG database with an *e*-value cutoff of 1e^−5^. The KEGG annotation was conducted using Diamond against the Kyoto Encyclopedia of Genes and Genomes (KEGG) database (http://www.genome.jp/keeg/) with an *e*-value cutoff of 1e^−5^. The pathogens were predicted with the pathogen–host interactions (PHI) database (http://www.phi-base.org/, version 4.4).

### Statistical Analysis

All data were checked for normality. The Wilcoxon rank-sum test in STAMP was used to seek for significant differences between groups, and the *p*-value was tested by Bonferroni correction. The linear discriminant analysis (LDA) effect size (LEfSe) method was used to identify bacterial taxa with significant difference among groups (http://huttenhower.sph.harvard.edu/galaxy/root?tool_id=lefse_upload). Principal component analysis (PCA) was calculated using weighted UniFrac distance metric in R software.

## Results and Discussions

Anthelmintic compounds are widely used in the equine populations because of their common parasitic infections. Anthelmintic treatment will gravely harm the animal's health if left uncontrolled. Previous studies showed that the composition and structure of the intestinal microbial community in horses could be changed upon the treatment of anthelmintics (23–25, 38). However, these studies lacked a control group of horses that were free of parasites, hence making it difficult to identify if the changes were due to the administered anthelmintics or due to the removal of parasites. Then, Kunz et al. (14) conducted an experiment to investigate how the intestinal microbes of uninfected horses changed under the administration of anthelmintic compounds. Their results did not show large-scale changes in the intestinal microbial community observed in infected horses treated with anthelmintics. Thus, changes in intestinal microbes of horses following anthelmintic treatment are mainly associated with the removal of parasites.

Horse botflies are the main concern for the wild Przewalski's horses in Xinjiang. Ivermectin is administered to the horses once a year in winter to control their parasitic infestation. Hence, understanding how these regular horse botfly expulsion treatments would impact the intestinal microbiota of the horses becomes important, as the microbiota plays an important role in influencing the host metabolism, immunity, speciation, and many other functions (39–44). However, previous studies were limited to studying only the bacterial community based on 16S rRNA sequencing, thus lacking information on other members such as archaea, fungi, and virus of the microbiota, and analysis of its functions.

### Differences in Composition of Intestinal Microbial Community in FATPHs and PATPHs

After quality control, a total of 437,143,618 reads (FATPHs: 222,498,384; PATPHs: 214,645,234) and 3,738,967 contigs (FATPHs: 1,932,396; PATPHs: 1,806,571) were obtained from all samples with an insert size of 565 bp and length of 150 bp (Table 1). The average values of N50 and N90 were 697 ± 73 bp and 350 ± 6 bp, respectively (Table 1). When comparing the reads with the database by the best-hit annotation method, five domains including archaea, bacteria, eukaryota, virus, and unclassified were identified. It was shown that most of the reads were annotated with bacteria, which accounted for 97.79% in FATPHs and 96.76% in PATPHs. Meanwhile, archaea, eukaryota, and virus occupied, respectively 1.07, 0.46, and 0.59% in FATPHs, and, respectively 0.87, 1.85, and 0.30% in PATPHs. Classification of the domains into each taxonomic unit showed that FATPHs had 8 kingdoms, 134 phyla, 263 classes, 579 orders, 1,052 families, 2,775 genera, and 12,677 species and correspondingly PATPHs had 8, 134, 267, 591, 1,081, 2,824, and 12,729, respectively. Both FATPHs and PATPHs had the same numbers of domains, kingdom, and phylum. Anthelmintic treatment reduced the microbial richness below the class level.

**Table 1:** Sequencing information of the six samples.

**Samples**	**Raw reads**	**Raw base (bp)**	**Contigs**	**Contigs bases (bp)**	**N50 (bp)**	**N90 (bp)**
FATPH1	69,471,868	10,490,252,068	548,642	376,741,716	721	347
FATPH3	72,653,294	10,970,647,394	682,192	474,946,359	734	353
FATPH6	72,520,072	10,950,530,872	701,562	513,945,911	791	358
PATPH1	74,061,672	11,183,312,472	552,710	358,478,060	668	345
PATPH3	70,389,906	10,628,875,806	624,273	362,638,296	574	342
PATPH6	78,046,806	11,785,067,706	629,588	426,246,469	692	353

#### Archaea

**Figure 1:**
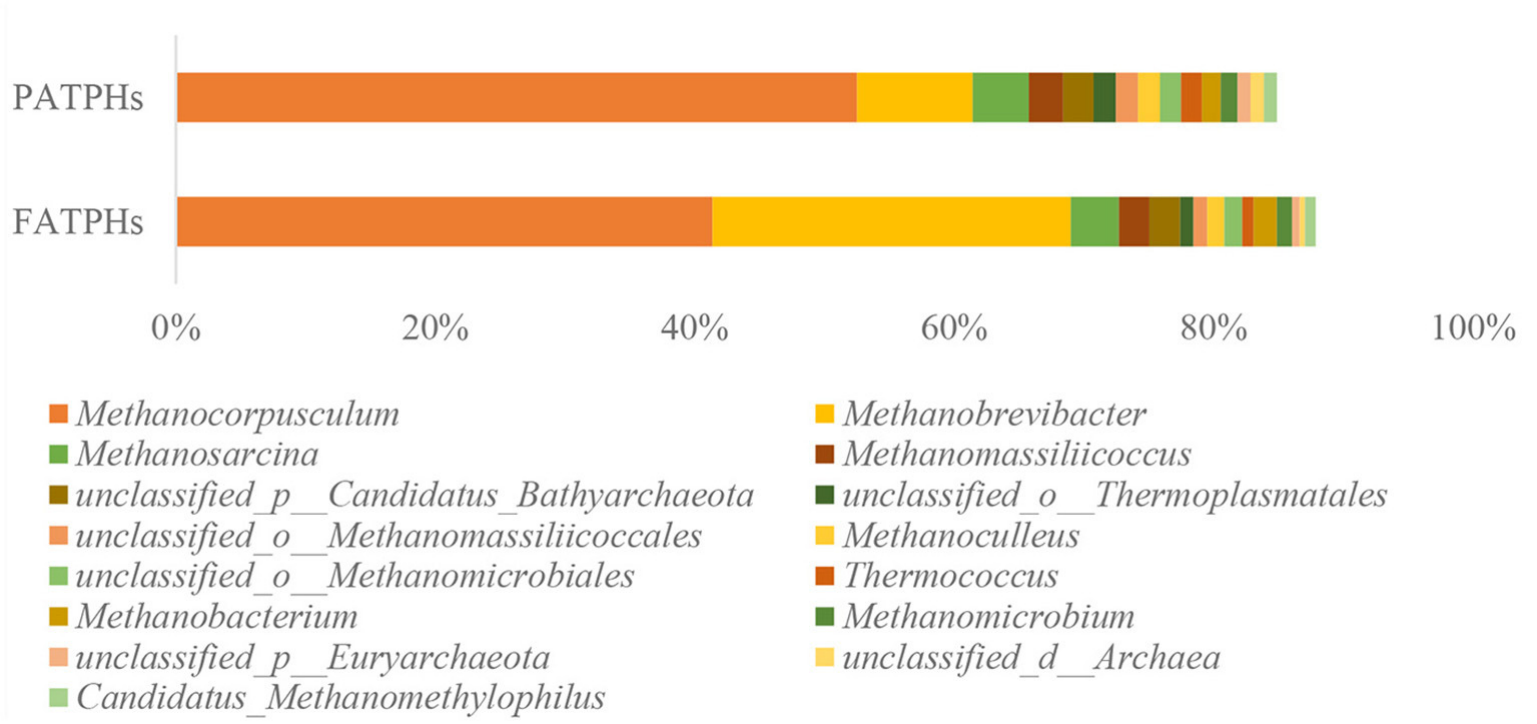
Relative abundances of archaea genera ranked by one and zero orders of magnitude in PATPHs shown together with relative abundances of the corresponding genera in FATPHs.

In archaea, the dominant phylum was *Euryarchaeota* in both FATPHs and PATPHs, accounting for more than 94% (FATPHs: 95.84%; PATPHs: 94.33%). The *Euryarchaeota* contains most species of the archaea, including methanogens that are often found in animal guts (45, 46). The top genus in PATPHs was *Methanocorpusculum* (52.52%), followed by *Methanobrevibacter* (8.93%), *Methanosarcina* (4.32%), *Methanomassiliicoccus* (2.65%), and unclassified_p_Candidatus_*Bathyarchaeota* (2.32%) (Figure 1). Compared with those in FATPHs, the abundance of *Methanocorpusculum* showed a decreasing trend (11.15%↓, *p* = 0.663) and that of *Methanobrevibacter* showed an increasing trend (18.69%↑, *p* = 0.081) after anthelmintic treatment. However, other top genera did not vary much. It is known that both *Methanocorpusculum* and *Methanobrevibacter* are methanogens that promote fermentation of carbohydrates and produce methane in the gut of mammals (47, 48). *Methanobrevibacter* spp. were the dominant methanogens found in the guts of goats and dairy cows (49–51). The abundance of *Methanocorpusculum* spp. showed an increasing trend in horses fed with forage (52).

**Figure 2:**
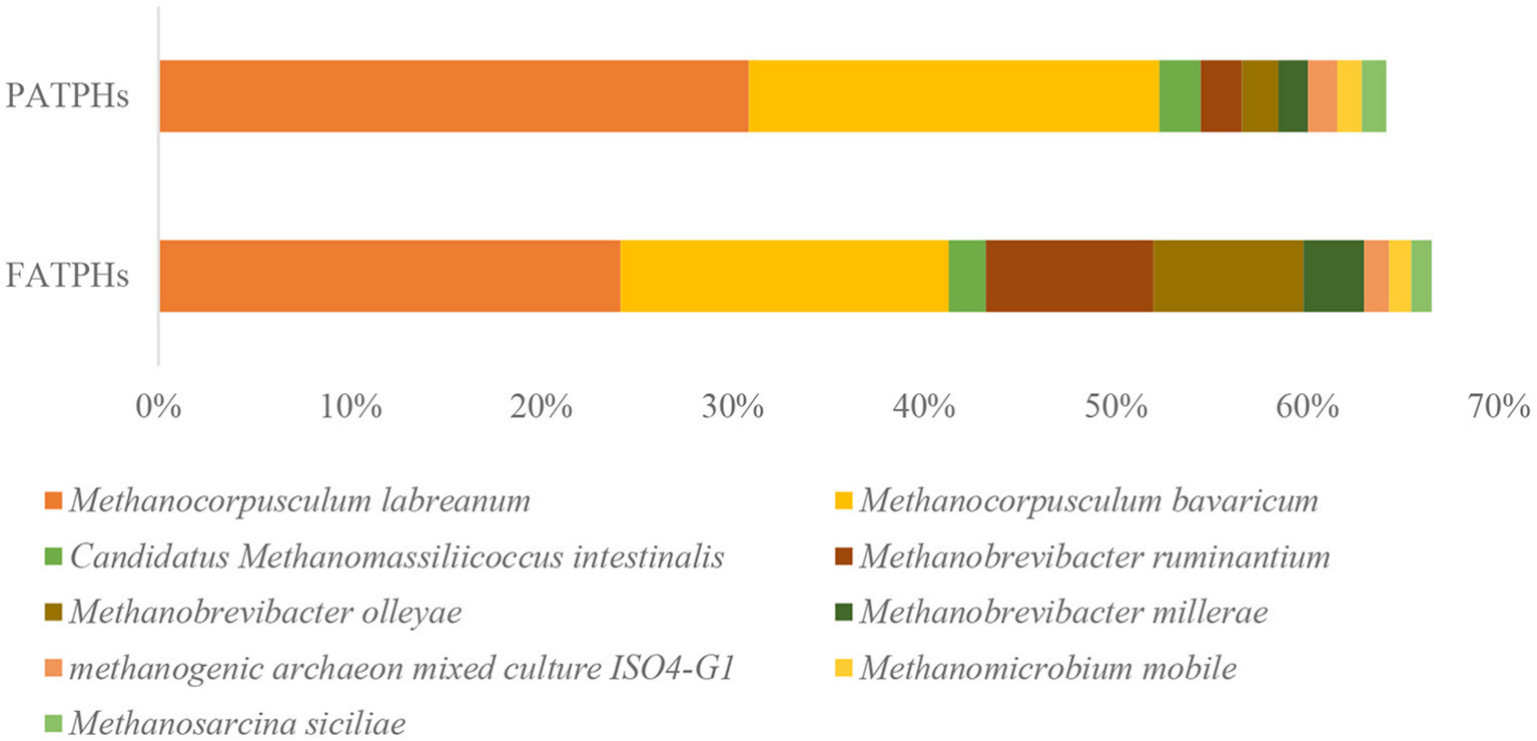
Relative abundances of archaea species ranked by one and zero orders of magnitude in PATPHs shown together with relative abundances of the corresponding species in FATPHs.

The use of metagenomics can classify the microbes into species level (53), which 16S rRNA sequencing cannot. The present metagenomics results showed that the species of *Methanocorpusculum labreanum* and *Methanocorpusculum bavaricum* dominated in both of the FATPHs and PATPHs. Their relative abundances were 24.15% (*Methanocorpusculum labreanum*) and 17.13% (*Methanocorpusculum bavaricum*), respectively in FATPHs and 30.85 and 21.45%, respectively in PATPHs. Both species showed a decreasing trend following anthelmintic treatment (*p* = 0.663) (Figure 2). Furthermore, there were seven species with relative abundances at zero order of magnitude in FATPHs and PATPHs (Figure 2). Among them, *Methanobrevibacter ruminantium* (6.63%↑, *p* = 0.081) and *Methanobrevibacter olleyae* (5.94%↑, *p* = 0.081) had a significant increasing trend following anthelmintic treatment. This was consistent with the findings in sheep by Moon et al. (54), who indicated that an increase in relative abundance of *Methanobrevibacter ruminantium* was associated with anthelmintic treatment.

#### Bacteria

A total of 82 bacterial phyla were detected in FATPHs and PATPHs, which is far more than the 23 phyla identified with 16S rRNA sequencing, referencing with the 16S rRNA sequencing results by Hu et al. (4), who did not find the presence of the *Gemmatimonadetes* phylum in FATPHs, but only a small 0.0012% in PATPHs. Results of the present metagenomic sequencing showed a successful detection of the *Gemmatimonadetes* in both FATPHs and PATPHs with relative abundances (percentage of read number in bacterial community) at 0.018 and 0.023%, respectively.

**Figure 3:**
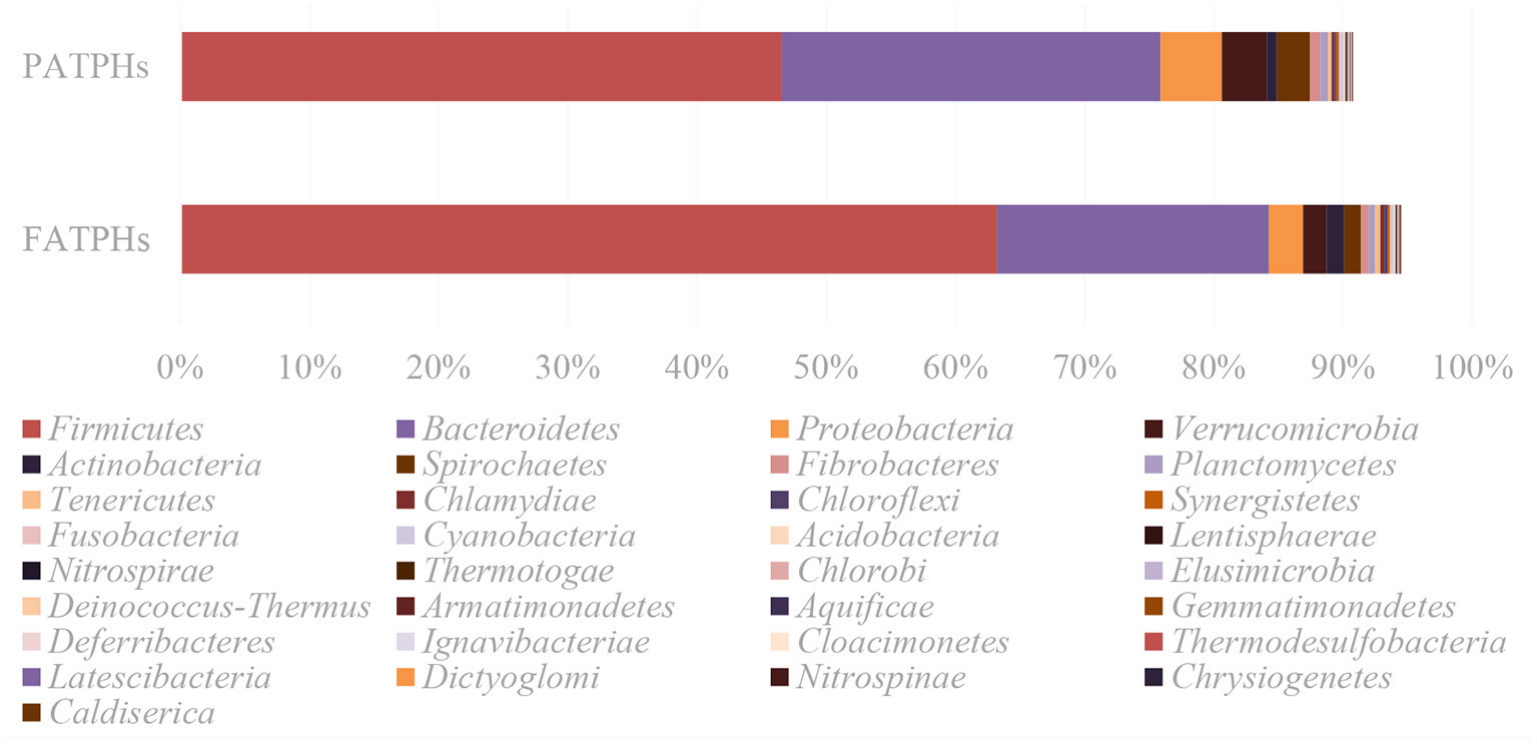
Relative abundances of the well classified bacterial phyla in FATPHs and PATPHs.

Overall, results of the metagenomic sequencing showed that the prevalent phyla were *Firmicutes* (FATPHs: 63.22%; PATPHs: 46.55%) and *Bacteroidetes* (FATPHs: 21.04%; PATPHs: 29.33%), which are commonly found in the guts of mammals (4, 18, 55, 56). It is noted that there were 49 phyla identified as unclassified bacteria (1 phylum), candidate division (9 phyla), and Candidatus phylum (39 phyla), with a total relative abundance of 5.55% in FATPHs and 9.26% in PATPHs. Meanwhile, comparison of the relative abundances of the 33 well-classified bacterial phyla in FATPHs and PATPHs showed that anthelmintic treatment reduced the relative abundances of 27 phyla and increased those of 6 phyla (Figure 3).

Moreover, there were five phyla of *Epsilonbacteraeota, Kiritimatiellaeota, Patescibacteria*, WPS-2, and unclassified_k__norank_d__Bacteria identified in 16S rRNA sequencing, but not found in metagenomic sequencing. At present, the main methods for studying microbial community based on high-throughput sequencing platform are marker gene amplicon (16S rRNA, 18S rRNA, ITS, etc.) and metagenomics (57). The advantages of 16S rRNA sequencing are fast, low cost, and easy result analysis, but biases associated with PCR amplification are inevitable. On the other hand, metagenomic sequencing is generally less affected by biases but more costly (58, 59). Thus, it is necessary to choose more than one sequencing method in studying one single sample.

**Figure 4:**
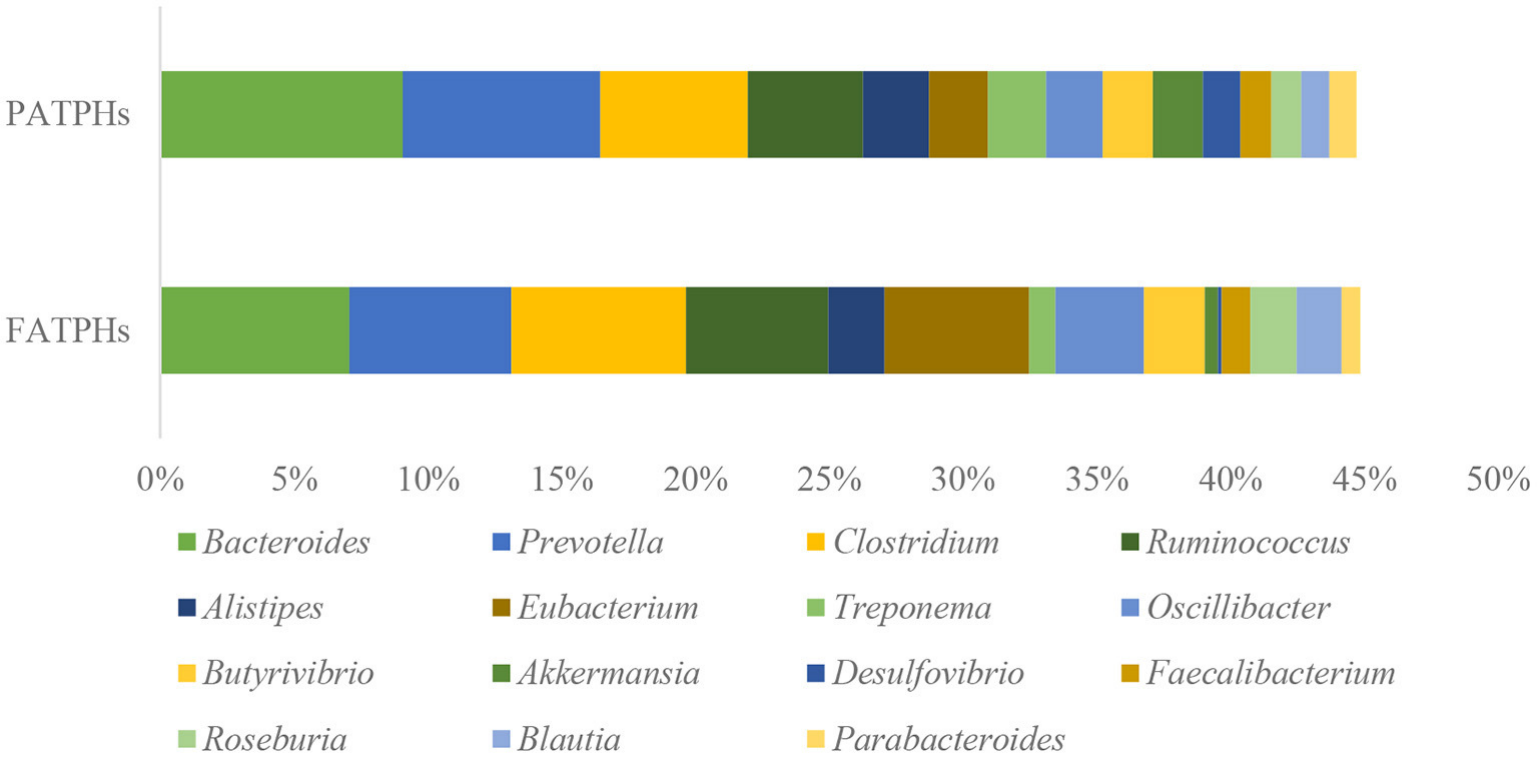
Top relative abundances of the well-classified bacterial genera ranked by zero order of magnitude in PATPHs shown together with the relative abundances of the corresponding genera in FATPHs.

At the genus level, the top three genera in PATPHs with relative abundance higher than 5% were *Bacteroides* (9.06%), *Prevotella* (7.39%), and *Clostridium* (5.52%) (Figure 4). Compared with the corresponding genera in FATPHs, *Clostridium* exhibited an increasing trend (FATPHs: 6.53%, *p* = 0.383) and *Bacteroides* (FATPHs: 7.07%, *p* = 0.663) and *Prevotella* (FATPHs: 6.05%, *p* = 0.383) showed a decreasing trend after anthelmintic treatment. Ramanan et al. (60) demonstrated that deworming treatment for mice would change their gut *Clostridium* and *Bacteroides* levels. Meanwhile, a significant abundance decrease of the *Bacteroides* was found in sika deer after anthelmintic treatment (30). The association between *Bacteroidetes* and IL-10, a key anti-inflammatory cytokine involved in the induction of immune suppression, could be established after anthelmintic treatment (61). *Clostridia* were known to facilitate the host immune responses due to their production of short-chain fatty acids including butyrate with anti-inflammatory properties (62, 63). Mice that were infected with *Trichuris muris* experienced a decline in *Prevotella* after the removal of the infection (64). A study indicated that the changed abundance of *Prevotella* could drive Th17 immune responses, which were associated with the occurrence and development of many inflammatory and autoimmune diseases (65). In addition, among the other genera identified in this study with relative abundance at zero order of magnitude, *Eubacterium* exhibited the largest increasing trend (3.21%↑, *p* = 0.081) following anthelmintic treatment. The increase of *Eubacterium* after deworming was consistent with a study conducted in humans infected by *Opisthorchis felineus* (66). *Eubacterium* was found related with the physiology of horses, negatively correlated with salivary cortisol levels, but positively correlated with N-butyrate production (67).

Indeed, disruption of the intestinal microbes by parasite infestation has the capacity to modify the host's immune regulatory system (68). For example, a type 2 immune response to parasitic infection of *Nippostrongylus brasiliensis* in mice was related to the altered intestinal microbial community, especially the segmented filamentous bacteria (69). Thus, it is suspected that anthelmintic treatment of the horse botfly infection could moderate the immune responses of the Przewalski's horses by shifting their relative abundances of *Bacteroides, Clostridium, Eubacterium*, and *Prevotella*. On the other hand, *Bacteroides* and *Prevotella* were associated with plant-rich diets, which played an important role in the breakdown of indigestible fibers (70). Combined with the large number of methanogen archaea identified, anthelmintic treatment may be capable of altering the digestive ability of the Przewalski's horses.

Referencing to previous studies conducted with parasite-infected horses based on 16S rRNA sequencing, anthelmintic treatment could change the relative abundances of *Streptococcus, Lactobacillus* (4), *Adlercreutzia* (22), and *Acinetobacter* (24). Metagenomic analysis in the present study already reveals additional effects of anthelmintic treatment on the intestinal bacterial community.

**Figure 5:**
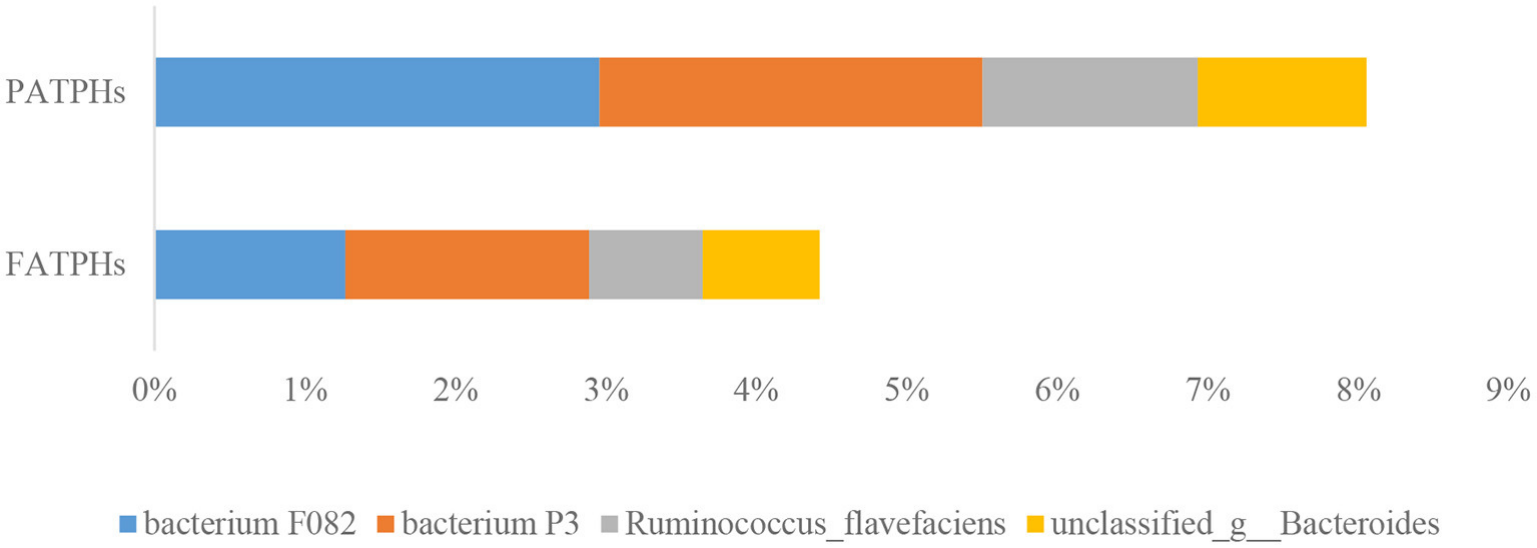
Relative abundances of top four bacterial species in PATPHs and their corresponding species in FATPHs.

At the species level, a total of 10,453 species were identified in FATPHs, and 10,421 in PATPHs, indicating a slight increase in bacterial species following anthelmintic treatment. All annotated species had relative abundances at zero order of magnitude or lower. The top four species in PATPHs were bacterium F082 (2.96%), bacterium P3 (2.55%), *Ruminococcus flavefaciens* (1.43%), and unclassified_g__Bacteroides (1.12%) (Figure 5). Their corresponding relative abundances at 1.27% (*p* = 0.383), 1.62% (*p* = 0.383), 0.76% (*p* = 0.383), and 0.78% (*p* = 0.383), respectively, in FATPHs showed a decreasing trend after anthelmintic treatment.

#### Eukaryota

Eukaryota is rarely studied in animal intestinal microbes. Very limited published articles on profiling the intestinal eukaryotic community of different animals can be found. They were done on dogs (71), humans (72), shrimps (73), and sika deer (30). The present study is the first to determine the composition and structure of the eukaryotic community in equine animal.

**Figure 6:**
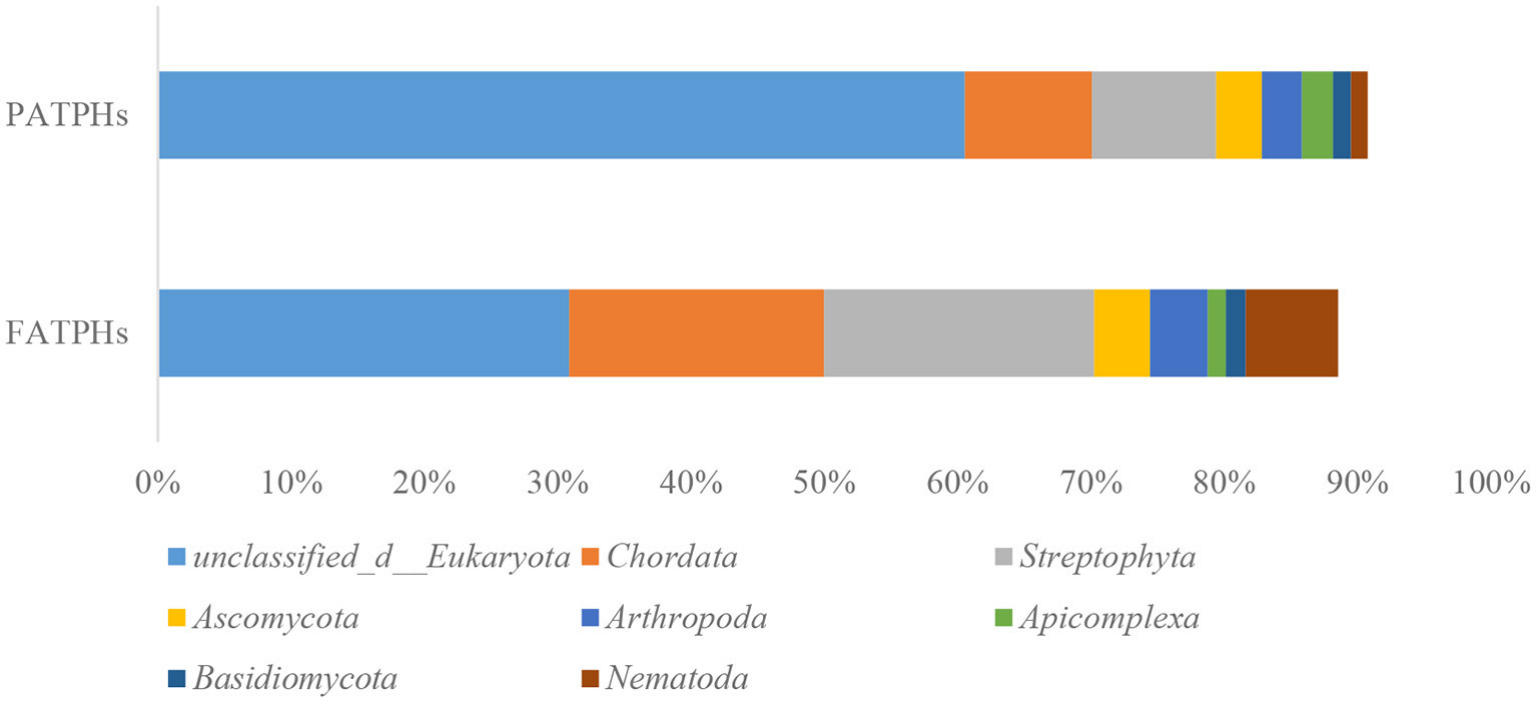
Relative abundances of eukaryota phyla ranked by one and zero orders of magnitude in PATPHs shown together with relative abundances of the corresponding phyla in FATPHs.

**Figure 7:**
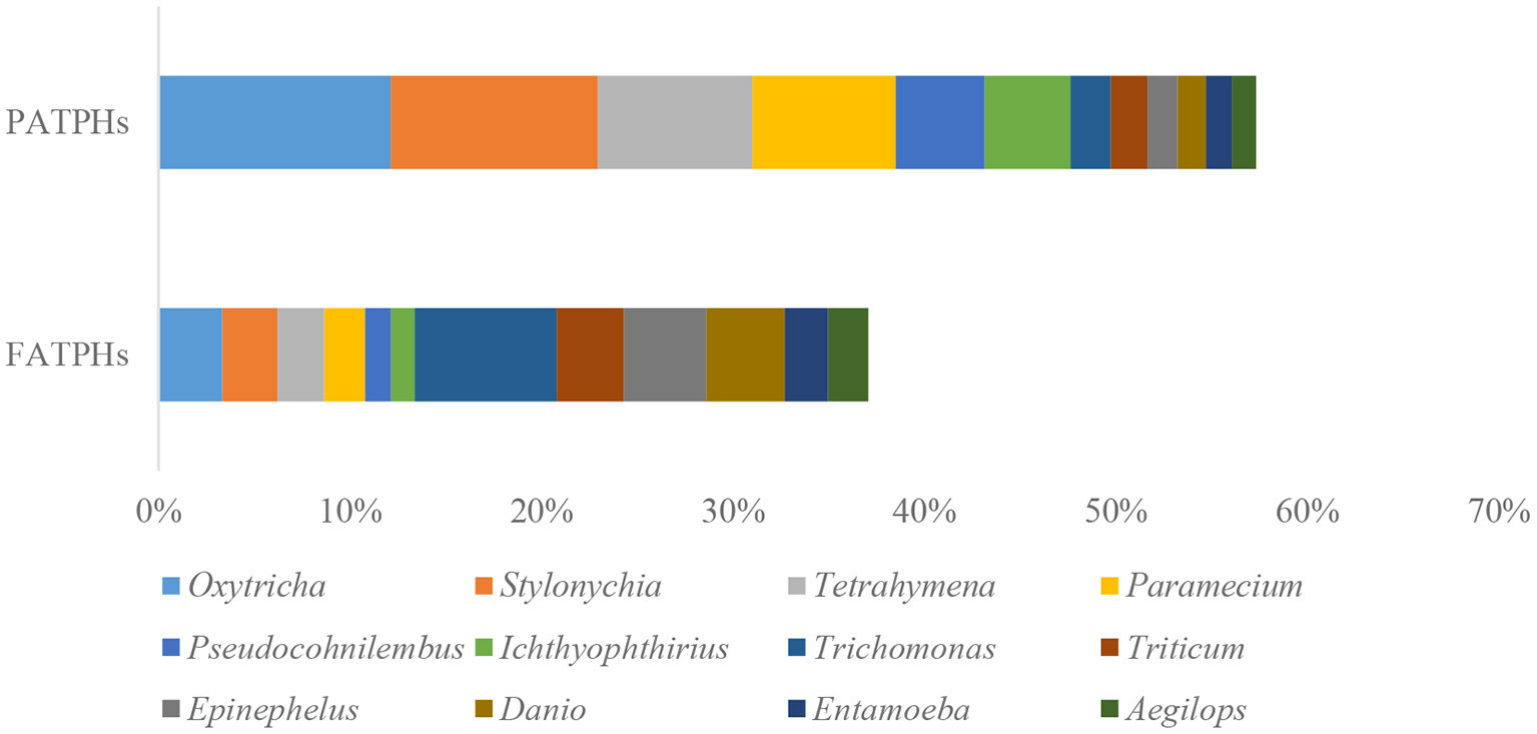
Relative abundances of eukaryota genera ranked by one and zero orders of magnitude in PATPHs shown together with relative abundances of the corresponding genera in FATPHs.

The top eukaryota phylum with relative abundance at one order of magnitude in PATPHs was unclassified_d__Eukaryota (60.53%) (Figure 6). It displayed a decreasing trend to 30.86% in FATPHs (*p* = 0.190) after anthelmintic treatment. With abundance at zero order of magnitude, PATPHs had seven phyla. They were *Chordata, Streptophyta, Ascomycota, Arthropoda, Apicomplexa, Basidiomycota*, and *Nematoda* (Figure 6). Among them, *Streptophyta* (10.97%↑, *p* = 0.190), *Chordata* (9.56%↑, *p* = 0.081), and *Nematoda* (5.67%↑, *p* = 0.663) showed large increasing trends in FATPHs after anthelmintic treatment. At the genus level, the relative abundances of all annotated genera were at one order of magnitude and lower. The 12 genera in PATPHs with abundances at one and zero orders of magnitude were *Oxytricha, Stylonychia, Tetrahymena, Paramecium, Pseudocohnilembus, Ichthyophthirius, Trichomonas, Triticum, Epinephelus, Danio, Entamoeba*, and *Aegilops* (Figure 7). Of them, five genera had their relative abundances shifted by more than 5% in FATPHs after anthelmintic treatment. They were *Oxytricha* (8.79%↓, *p* = 0.190), *Stylonychia* (7.95%↓, *p* = 0.190), *Tetrahymena* (5.62%↓, *p* = 0.190), *Paramecium* (5.36%↓, *p* = 0.190), and *Trichomonas* (5.31%↑, *p* = 0.383). A study of interventional treatment with probiotics and a low-fat diet on humans showed that the levels of the abovementioned genera except *Streptophyta* were reduced (74). Therefore, anthelmintic treatment may affect the digestion of the treated Przewalski's horses. Note that characteristics of the other eukaryota genera identified have not yet been studied.

Finally, there were 1,139 species and 1,034 species identified in PATPHs and FATPHs, respectively. Two species, *Oxytricha trifallax* (12.12%) and *Stylonychia lemnae* (10.83%), in PATPHs had relative abundances larger than 10%. Their abundances were reduced by an order of magnitude to 3.33% (*p* = 0.190) and 2.88% (*p* = 0.190), respectively, in FATPHs after anthelmintic treatment.

#### Virus

So far, the intestinal viral community was just done in dogs, cats, and humans (75, 76). This is the first study to determine the composition and structure of the viral community in equines.

There was only one viral phylum unclassified_d__Viruses identified in this study. Under this phylum, 70 genera were found in FATPHs and 69 in PATPHs. Eleven of the identified genera were in the unclassified group, accounting for 75.19% in FATPHs and 79.06% in PATPHs. The top three genera in PATPHs with relative abundances at one order of magnitude were unclassified_d__Viruses (26.91%), unclassified_f__*Siphoviridae* (25.32%), and unclassified_f__*Myoviridae* (17.48%). Comparing the abundances of theirs in FATPHs after anthelmintic treatment, unclassified_d__Viruses (29.33%, *p* = 0.081) showed an increasing trend while unclassified_f__*Siphoviridae* (23.76%, *p* = 1.000) and unclassified_f__*Myoviridae* (9.41%, *p* = 0.663) exhibited a decreasing trend. The large number of viruses annotated to unclassified indicates that there is a great opportunity to find new species in the equine gut, which is an unexplored habitat.

At the species level, 681 and 656 viruses were found in FATPHs and PATPHs, respectively. The top 10 abundant species consisted of one unidentified phage and nine uncultured viruses, making up 25.53 and 21.81% of the community in FATPHs and PATPHs, respectively. It is noted that most of viral species were classified into phage with 128 phages in FATPHs and 96 in PATPHs, occupying 71.49% of relative abundance in FATPHs and 72.98% in PATPHs, respectively. The relative abundance of phage changed more than other viral species after anthelmintic treatment.

### Pathogens in FATPHs and PATPHs

**Figure 8:**
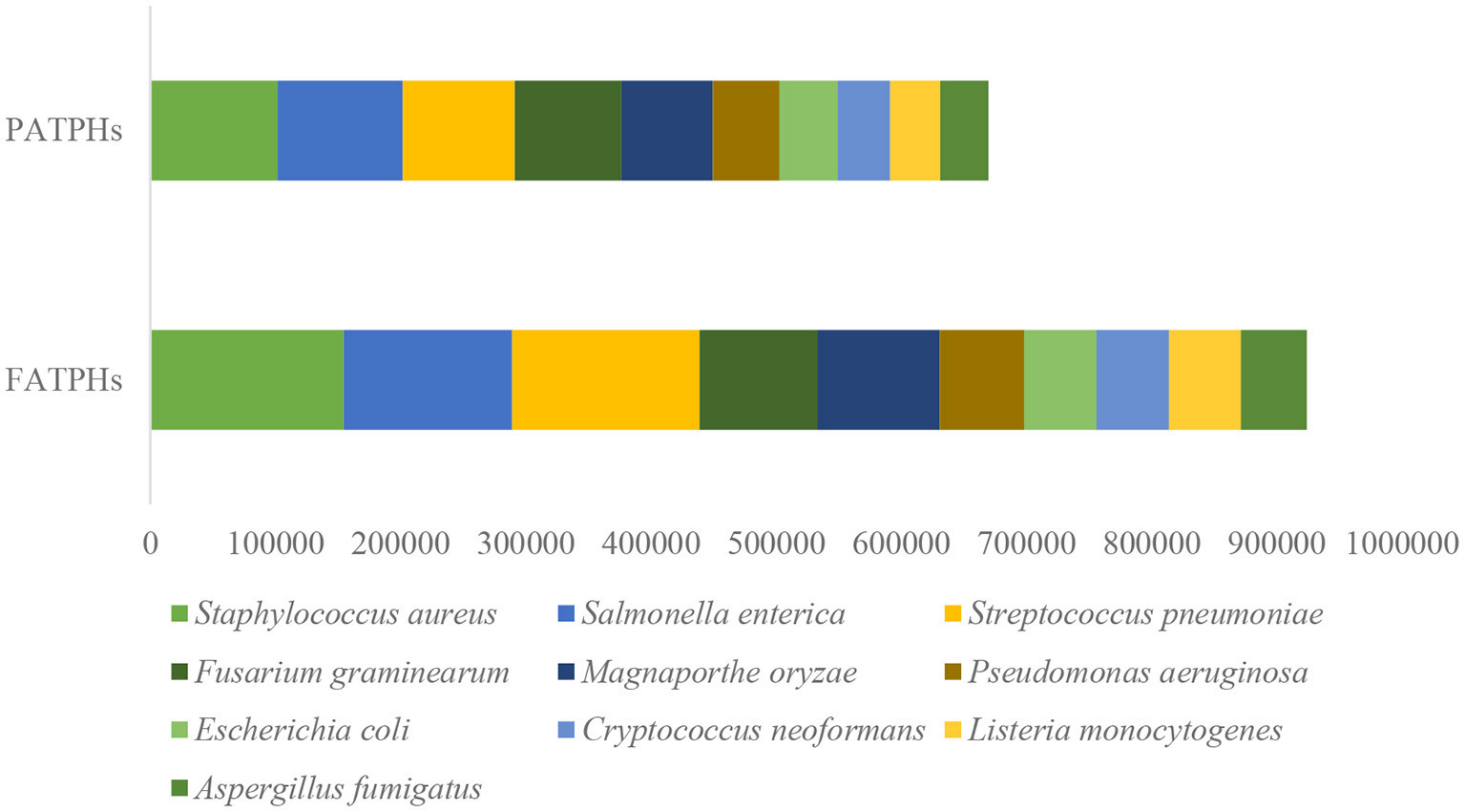
Top 10 pathogens ranked by their gene abundances in PATPHs shown together with gene abundances of the corresponding pathogens in FATPHs.

There were totally 187 pathogens of 108 genera found in the samples when comparing the identified reads to the PHI database (Supplementary Table 1). The anthelmintic treatment increased the abundances of 128 pathogens and reduced those of 59 others. The top 10 pathogens based on gene abundance in PATPHs were *Staphylococcus aureus* (Bacteria), *Salmonella enterica* (Bacteria), *Streptococcus pneumoniae* (Bacteria), *Fusarium graminearum* (Eukaryota), *Magnaporthe oryzae* (Eukaryota), *Pseudomonas aeruginosa* (Bacteria), *Escherichia coli* (Bacteria), *Cryptococcus neoformans, Listeria monocytogenes* (Eukaryota), and *Aspergillus fumigatus* (Eukaryota) (Figure 8). Their abundances were all increased in FATPHs following anthelmintic treatment. For these pathogens, Eukaryota-related diseases have not been observed in equine animals. The methicillin-resistant *Staphylococcus aureus* (MRSA) is an emerging equine pathogen and is associated with a series of clinical diseases, such as septic arthritis, intravenous (jugular) catheter site infections, pneumonia cases, incisional infection, wound infection, mastitis, rhinitis, and body wall absces (77, 78). Horses infected by *Salmonella enterica* suffer from some clinical signs, such as fever, dehydration, diarrhea, colic, and septicemia (79, 80). Infection of *Streptococcus pneumoniae* will trigger immune response of the horse to accumulate leucocytes and cytokine (81). *Pseudomonas aeruginosa* is an opportunistic pathogen that is commonly recognized as a cause of endometritis in horses (82, 83). *Escherichia coli* are common commensal bacteria found in the intestinal tract of horses; they can cause diarrhea (83, 84). Casual usage of anthelmintic treatment poses a risk to the horses, known as colic (26). The common characteristics of an intestinal microbial community after antibiotic treatment include change of microbial diversity, increase of the colonization of pathogens, and development of antimicrobial resistance (85, 86). Hence, the removal of horse botflies through anthelmintic treatment raised the gene abundance of the top pathogens in the studied Przewalski's horses, which could increase their risk of sickness. However, this is limited by the small sample size used in the present study. Future repeated studies are needed for confirmation.

### Functional Analysis of Intestinal Microbiota Between FATPHs and PATPHs

It was shown that horse botfly expulsion by anthelmintic treatment for the studied Przewalski's horses altered their intestinal microbial community composition and structure. These changes in turn could affect the metabolism and physiology of the horses, such as microbial triggered immune responses (87), aid in regulation of energy metabolism (88, 89), and synthesis of the short-chain fatty acids or amino acids (90–92). In fact, it is increasingly recognized that the metabolism of animals is significantly affected by its intestinal microbes (93). Understanding the changes in intestinal microbial function is another key step toward clarifying the effects of anthelmintic treatment on horses. Metagenomic sequencing not only can characterize the gene content of a studied sample but also can predict the functional potential of its microbial community. The following sections present the metagenomic sequencing results on the intestinal microbial functions of the FATPHs and PATPHs.

#### COG Functional Annotation

**Figure 9:**
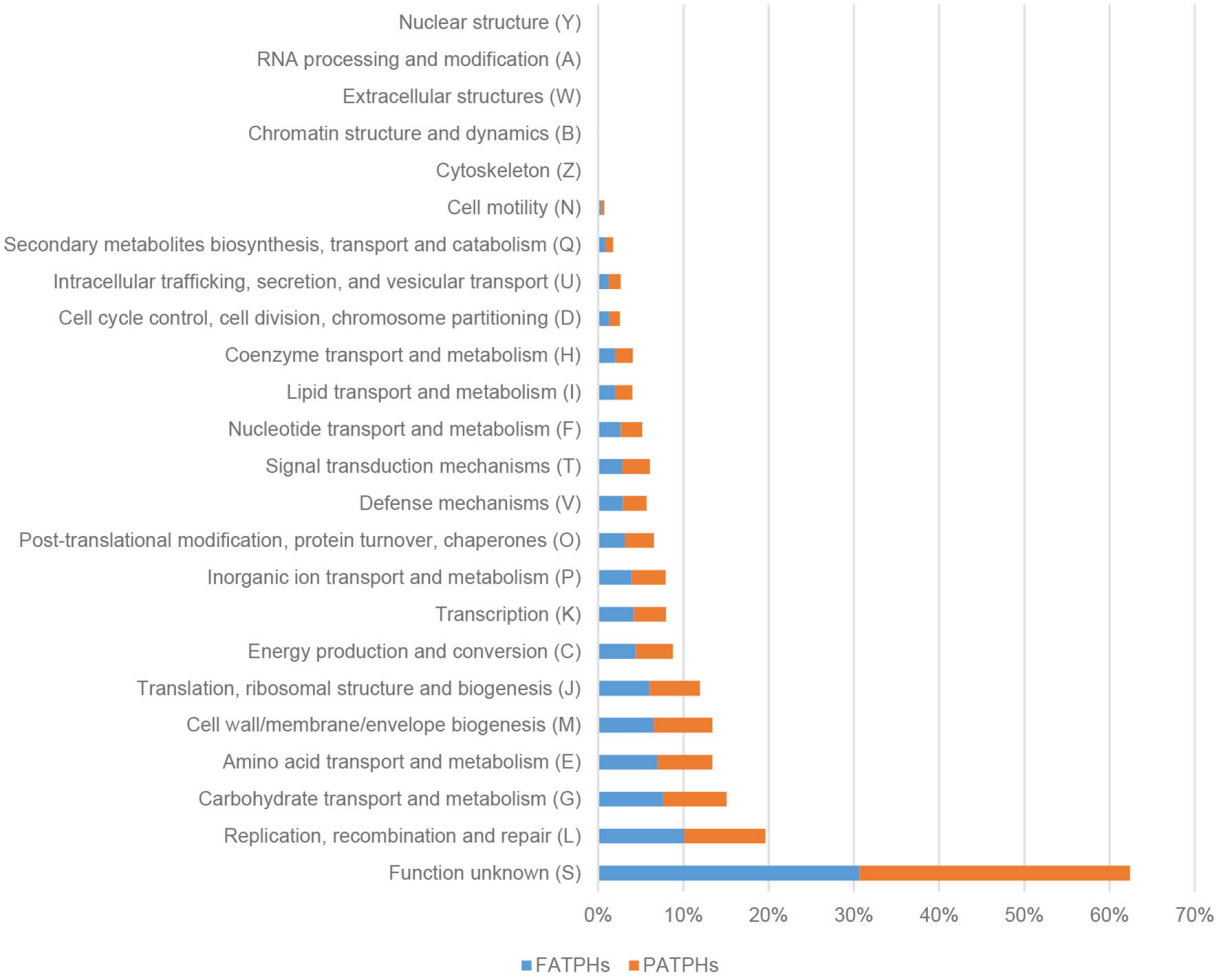
Distribution of COG functions in FATPHs and PATPHs. *E* ≤ 1e−5.

A total of 4,869,449 genes with a total length of 2,194,175,671 were identified from the six samples used in this study. There were 24 kinds of COG functions associated with cellular processes and signaling, information storage and processing, metabolism, and poor characterization found in FATPHs and PATPHs. The top function was Function unknown, which accounted for 30.67% in FATPHs and 31.72% in PATPHs (Figure 9). There were five functions with their percentages of read numbers over 5%. They were replication, recombination, and repair (FATPHs: 10.09%; PATPHs: 9.55%); carbohydrate transport and metabolism (FATPHs: 7.61%; PATPHs: 7.45%); amino acid transport and metabolism (FATPHs: 6.98%; PATPHs: 6.44%); cell wall/membrane/envelope biogenesis (FATPHs: 6.56%; PATPHs: 6.86%); and translation, ribosomal structure, and biogenesis (FATPHs: 6.07%; PATPHs: 5.87%) (Figure 9). Distribution of the function catalog was consistent with that of Tang et al. (55), who studied some captive and wild Przewalski's horses without any anthelmintic treatment. Their samples were similar to those of the PATPHs in the present study.

One important function of the intestinal microbes is metabolism, which converts carbohydrate and protein of dietary substrates to beneficial metabolites or alternative energy sources for the host (94). The intestinal microbes of the Przewalski's horses were found mainly to participate in carbohydrate and amino acid metabolism. The fermentation products of carbohydrate metabolism by microbes are short-chain fatty acids and gases. The three principal short-chain fatty acids detected in feces were acetate, butyrate, and propionate (95). Acetate plays an important role in regulating central appetite (96). Butyrate is capable of inducing the growth of cancer cells and colonic tumor cell lines, inhibiting mRNA expression and telomerase activity of cancer cells in human, enhancing memory recovery and formation, and preventing obesity in mice (97, 98). Propionate can be directly involved in portal-brain neural communication, induction of intestinal gluconeogenesis, and positive influence of the host's metabolism (99). Amino acids are key components of human and animal nutrition, which can regulate the intestinal bacterial community composition (100, 101).

For these two important functions of the intestinal microbiota, the top five species involved in carbohydrate transport and metabolism were *Ruminococcus flavefaciens, Clostridium* sp.CAG:413, *Faecalibacterium* sp.CAG:74, *Bacteroides* sp.CAG:1060, and *Fibrobacter succinogenes* in PATPHs, and *Streptococcus gallolyticus, Eubacterium* sp.CAG:581, *Oscillibacter* sp.CAG:241, *Oscillibacter* sp.ER4, and *Ruminococcus bromii* in FATPHs (Supplementary Figure 1). The top five species involved in amino acid transport and metabolism were *Oscillibacter* sp.ER4, *Ruminococcus flavefaciens, Fibrobacter succinogenes, Oscillibacter* sp.CAG:241, and *Clostridium* sp.CAG:413 in PATPHs, and *Oscillibacter* sp.ER4, *Streptococcus gallolyticus, Oscillibacter* sp.CAG:241, *Eubacterium* sp.CAG:581, and *Ruminococcus* sp.CAG:563 in FATPHs (Supplementary Figure 1). Although the genes annotated with metabolism did not change significantly after anthelmintic treatment, the pattern of functional species was completely different between FATPHs and PATPHs. Anthelmintic treatment had a displacement effect on the same functional microbes.

**Figure 10:**
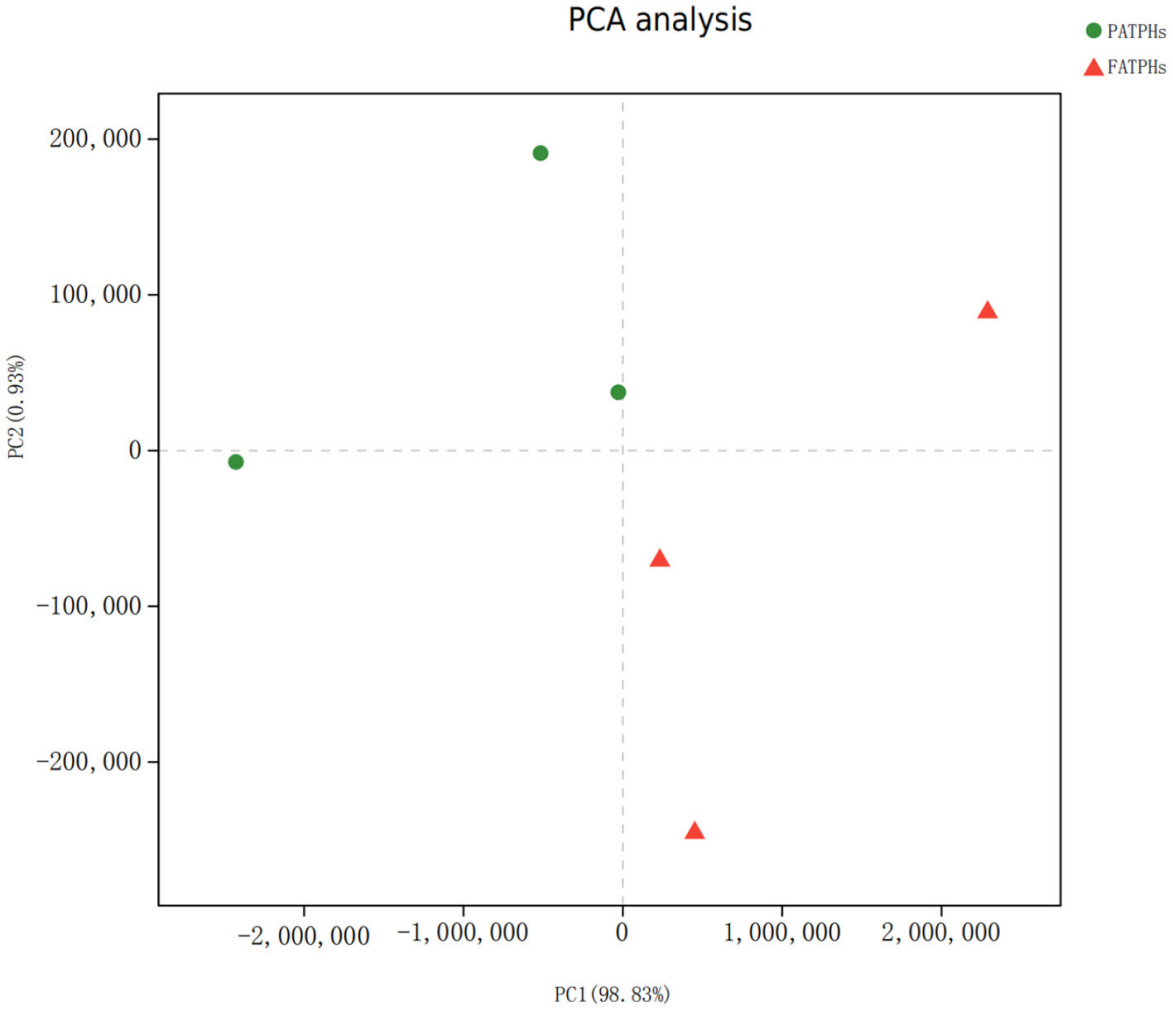
PCA analysis of FATPHs and PATPHs based on calculation of the numbers of reads annotated to COG functions. Both chart axes have accounted for 99.76% of variance. Red triangles represent samples of FATPHs, while green dots represent samples of PATPHs.

**Figure 11:**
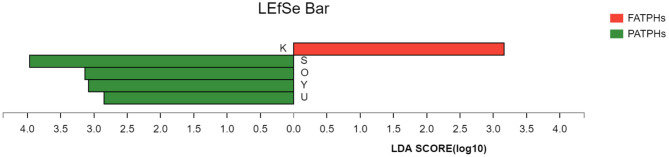
Cladogram showing the COG functions with significant differences between FATPHs and PATPHs. Functions with significant difference that have an LDA score >the threshold value of 3 are shown. K represents function of Transcription; S represents function of Function unknown; O represents function of Post-translational modification, protein turnover, and chaperones; Y represents function of Nuclear structure; and U represents function of Intracellular trafficking, secretion, and vesicular transport. The red bar represents samples of FATPHs, while green bars represent samples of PATPHs.

PCA analysis showed that FATPHs and PATPHs can be separated based on the COG functional genes (Figure 10). It means that anthelmintic treatment changed the functions of the intestinal microbes. LEfSe analysis depicted that four functions showed significant differences between FATPHs and PATPHs (Figure 11). Transcription (LDA = 3.16, *p* = 0.05) showed significant effects on FATPHs while Function unknown (LDA = 3.97, *p* = 0.05), post-translational modification, protein turnover, chaperones (LDA = 3.14, *p* = 0.05), Nuclear structure (LDA = 3.08, *p* = 0.05), and intracellular trafficking, secretion, and vesicular transport (LDA = 2.85, *p* = 0.05) had significant effects on PATPHs. These results indicated that the functional differences of intestinal microbes resulted from some essential cellular processes rather than metabolism.

#### KEGG Pathway Annotation

**Figure 12:**
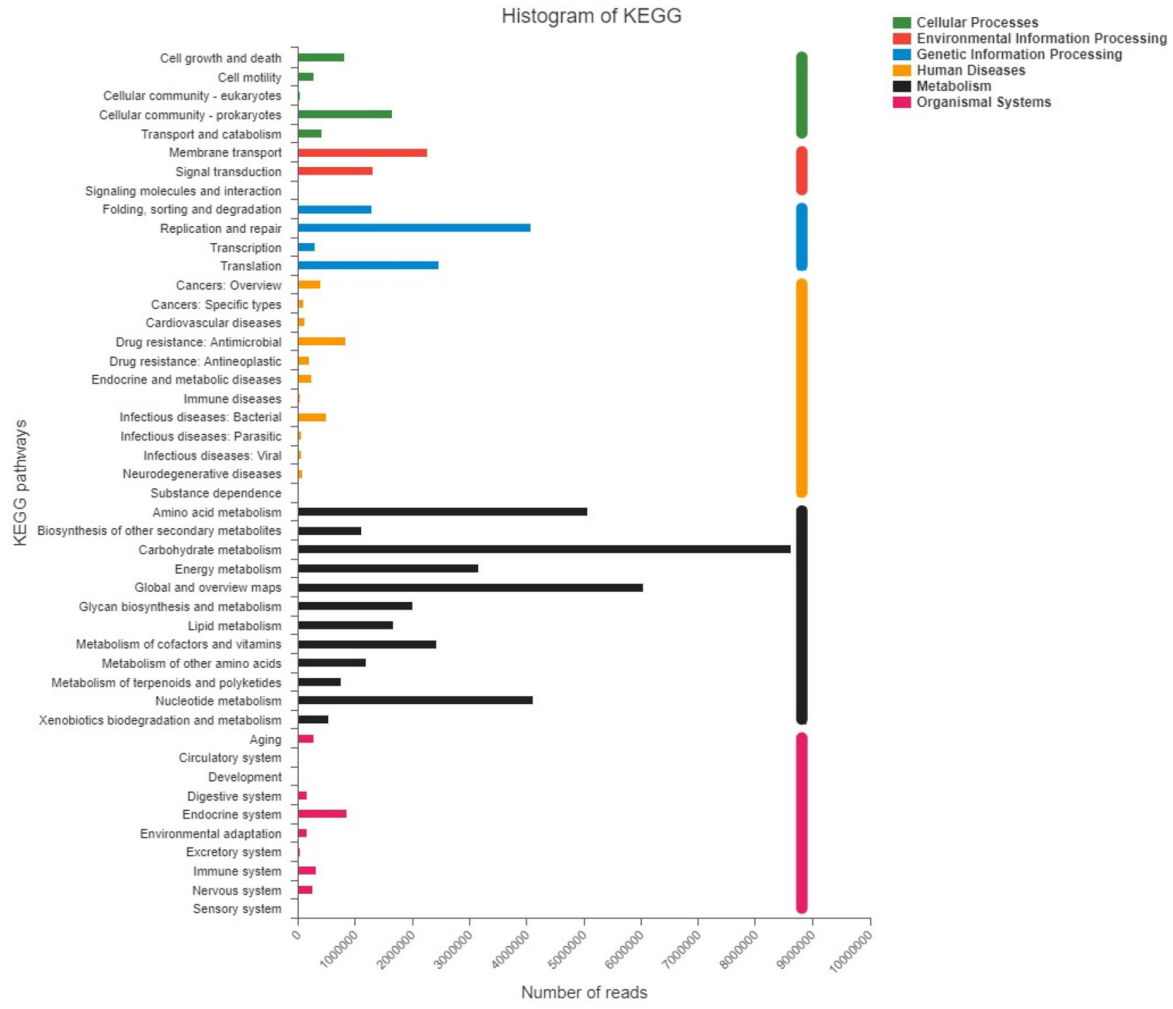
KEGG pathways at Levels 1 and 2 found in FATPHs and PATPHs. *E* ≤ 1e−5.

The reads were annotated with six KEGG pathways at level 1, including Cellular processes (FATPHs: 5.45%; PATPHs: 5.96%), Environmental information processing (FATPHs: 6.28%; PATPHs: 6.41%), Genetic information processing (FATPHs: 14.67%; PATPHs: 14.00%), Human diseases (FATPHs: 4.38%; PATPHs: 5.17%), Metabolism (FATPHs: 66.02%; PATPHs: 53.84%), and Organismal systems (FATPHs: 3.20%; PATPHs: 4.62%) (Figure 12). Over 50% Level 1 KEGG pathways in FATPHs and PATPHs were Metabolism, consistent with results of COG function annotation. At level two, a total of 46 pathways were found and the top 3 belonged to Metabolism, which were Carbohydrate metabolism (FATPHs: 15.63%; PATPHs: 14.83%), Global and overview maps (FATPHs: 10.98%; PATPHs: 10.30%), and Amino acid metabolism (FATPHs: 9.14%; PATPHs: 8.76%). Carbohydrate and amino acid still represent the dominant function of the intestinal microbiota of the Przewalski's horses. In the KEGG pathway, the top five species involved in Carbohydrate metabolism were *Clostridium* sp.CAG:413, *Ruminococcus flavefaciens, Faecalibacterium* sp.CAG:74, *Bacteroides* sp. CAG:1060 and *Oscillibacter* sp. ER4 in PATPHs, and *Streptococcus gallolyticus, Eubacterium* sp.CAG:581, *Oscillibacter* sp.ER4, *Oscillibacter* sp.CAG:241, and *Clostridium* sp.CAG:413 in FATPHs (Supplementary Figure 2). For the Amino acid metabolism, the top five species contributors were *Oscillibacter* sp.ER4, *Ruminococcus flavefaciens, Fibrobacter succinogenes, Clostridium* sp.CAG:413, and *Bacteroides* sp.CAG:1060 in PATPHs, and *Oscillibacter* sp.ER4, *Streptococcus gallolyticus, Eubacteriu*m sp.CAG:581, *Ruminococcus* sp.CAG:563, and *Oscillibacter* sp.CAG:241 in FATPHs (Supplementary Figure 2). The results also suggested that anthelmintic treatment did not change the numbers of reads related to the pathways of metabolism but influenced the composition of the functional intestinal microbial community.

## Conclusions

In this study, the composition and structure of the complex intestinal microbial community of Przewalski's horses prior to and following anthelmintic treatment were identified by metagenomic sequencing for the first time. The pathogens were determined based on the gene information. The obtained sequences were mapped to known genes or pathways in COG and KEGG databases. The results indicated that anthelmintic treatment might have adverse effects on horses, which needs to be further confirmed; thus, optimization is suggested for this strategy of controlling parasite infections or search for alternative methods in the future.

## Data Availability Statement

The datasets presented in this study can be found in online repositories. The names of the repository/repositories and accession number(s) can be found in the article/Supplementary Material.

## Author Contributions

DH, JY, YQ, BL, and KL conceived the experiments. DH and KL undertook the sampling work and conducted the experiments. DH, KL, and KMM analyzed the results and wrote the manuscript. All the authors read and approved the final manuscript.

## Conflict of Interest

The authors declare that the research was conducted in the absence of any commercial or financial relationships that could be construed as a potential conflict of interest.

## Publisher's Note

All claims expressed in this article are solely those of the authors and do not necessarily represent those of their affiliated organizations, or those of the publisher, the editors and the reviewers. Any product that may be evaluated in this article, or claim that may be made by its manufacturer, is not guaranteed or endorsed by the publisher.
