# Supplementary figures and images for: Metagenomic Analysis of Fecal Archaea, Bacteria, Eukaryota, and Virus in Przewalski's Horses Following Anthelmintic Treatment
Source: Front Vet Sci. 2021 Aug 18;8:708512. doi: 10.3389/fvets.2021.708512 (PMC8416479; doi:10.3389/fvets.2021.708512)

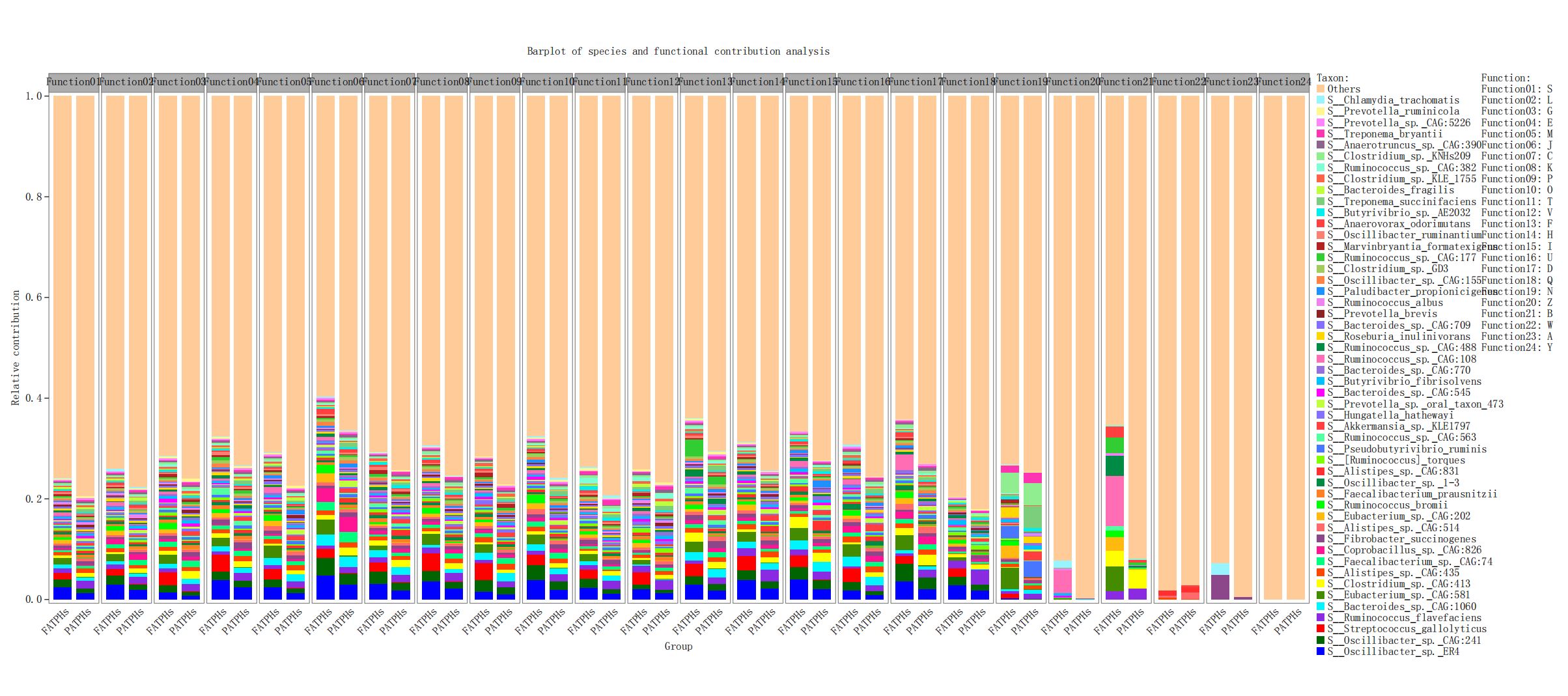

Supplement: Supplementary file 2 [file Image_1.JPEG]

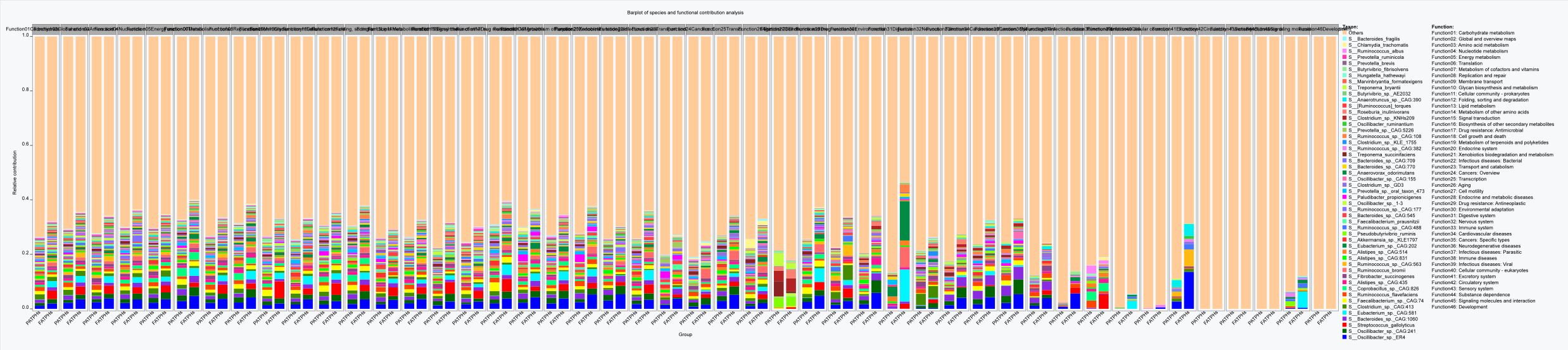

Supplement: Supplementary file 3 [file Image_2.JPEG]
